# Enteric nervous system and inflammatory bowel disease

**DOI:** 10.1093/gastro/goag005

**Published:** 2026-02-13

**Authors:** Shixian Wang, Yufeng Wang, Ji Miao, Xiaolong Zheng, Wei Ge, Gang Chen, Yi Yin

**Affiliations:** Division of Colorectal Surgery, Department of General Surgery, Nanjing Drum Tower Hospital, Affiliated Hospital of Nanjing University Medical School, Nanjing, Jiangsu, P. R. China; Department of General Practice, The Second Affiliated Hospital of Nanjing Medical University, Nanjing, Jiangsu, P. R. China; Division of Colorectal Surgery, Department of General Surgery, Nanjing Drum Tower Hospital, Affiliated Hospital of Nanjing University Medical School, Nanjing, Jiangsu, P. R. China; Division of Gastric Surgery, Department of General Surgery, Nanjing Drum Tower Hospital, Affiliated Hospital of Medical School, Nanjing University, Nanjing, Jiangsu, P. R. China; Division of Colorectal Surgery, Department of General Surgery, Nanjing Drum Tower Hospital, Affiliated Hospital of Nanjing University Medical School, Nanjing, Jiangsu, P. R. China; Division of Colorectal Surgery, Department of General Surgery, Nanjing Drum Tower Hospital, Affiliated Hospital of Nanjing University Medical School, Nanjing, Jiangsu, P. R. China; Division of Colorectal Surgery, Department of General Surgery, Nanjing Drum Tower Hospital, Affiliated Hospital of Nanjing University Medical School, Nanjing, Jiangsu, P. R. China

**Keywords:** enteric nervous system, inflammatory bowel disease, colorectal cancer, neuro-immune cross talk, enteric neurons, enteric glial cells

## Abstract

The enteric nervous system (ENS), often termed the “second brain,” plays a pivotal role in regulating gastrointestinal functions and maintaining intestinal homeostasis. This review explores the intricate interactions between the ENS and inflammatory bowel disease (IBD), emphasizing how ENS dysfunction contributes to IBD pathogenesis. Key findings highlight that IBD patients exhibit enteric neuropathies, including heightened neural excitability, synaptic vulnerability, and diminished inhibitory signaling, which exacerbate intestinal inflammation and barrier dysfunction. Bidirectional communication between enteric neurons, glial cells, and immune cells is critical in modulating immune responses and inflammation. Enteric glial cells (EGCs) emerge as central regulators of gut homeostasis, influencing neuronal survival, immune cell activity, and mucosal integrity, while their dysfunction contributes to chronic inflammation and colorectal cancer progression. Experimental colitis revealed that neuro-immune crosstalk, mediated by neurotransmitters and cytokines, exerted both protective and pro-inflammatory effect on colitis. Furthermore, the ENS contributes to colorectal cancer through neurogenesis, perineural invasion, and paracrine interactions with tumor cells. Emerging therapies targeting ENS activity, such as electrical neuromodulation and neuromodulators, showed promising results in alleviating IBD symptoms by restoring neural-immune balance. However, studying the ENS poses challenges such as low abundance of neuronal cell and technical limitations, which necessitate advanced methodologies like spatial transcriptomics. This review underscores the ENS as a therapeutic frontier for IBD and colorectal cancer, urging interdisciplinary approaches to unravel its multifaceted roles in health and disease.

## Background

The enteric nervous system (ENS) is a sophisticated and expansive component of the peripheral nervous system that regulates the behavior of the gastrointestinal tract with a degree of independence of the central nervous system (CNS) [1–3]. Often referred to as the “second brain”, ENS is closely associated with digestive disorders, owing to its autonomy in controlling gastrointestinal motility, secretion, and blood flow [4, 5]. The efferent projections from the CNS target the skeletal motor or autonomic nervous system. The autonomic nervous system traditionally divided into three parts: the sympathetic, parasympathetic, and enteric nerves. In contrast to neurons in the sympathetic or parasympathetic ganglia, most enteric neurons do not receive direct innervation from the CNS, due to the independent operation of the ENS. The ENS comprises a complex lattice of neurons and glial cells arranged into functional units known as microcircuits [1]. These microcircuits encompass intrinsic primary afferent neurons capable of responding autonomously to local stimuli, allowing the ENS to process sensory information and coordinate motor responses without the involvement of the CNS. This unique organization allows the ENS to possess both sensory and motor properties, enabling its regulatory functions independently from the CNS. Despite its functional autonomy, the ENS engages in bidirectional communication with the CNS, enabling a constant exchange of information [3]. The signaling interplay between the ENS and CNS helps ensure that the gastrointestinal system functions in harmony with the overall body physiology, with the gut influencing brain function and vice versa.

**Figure 1:**
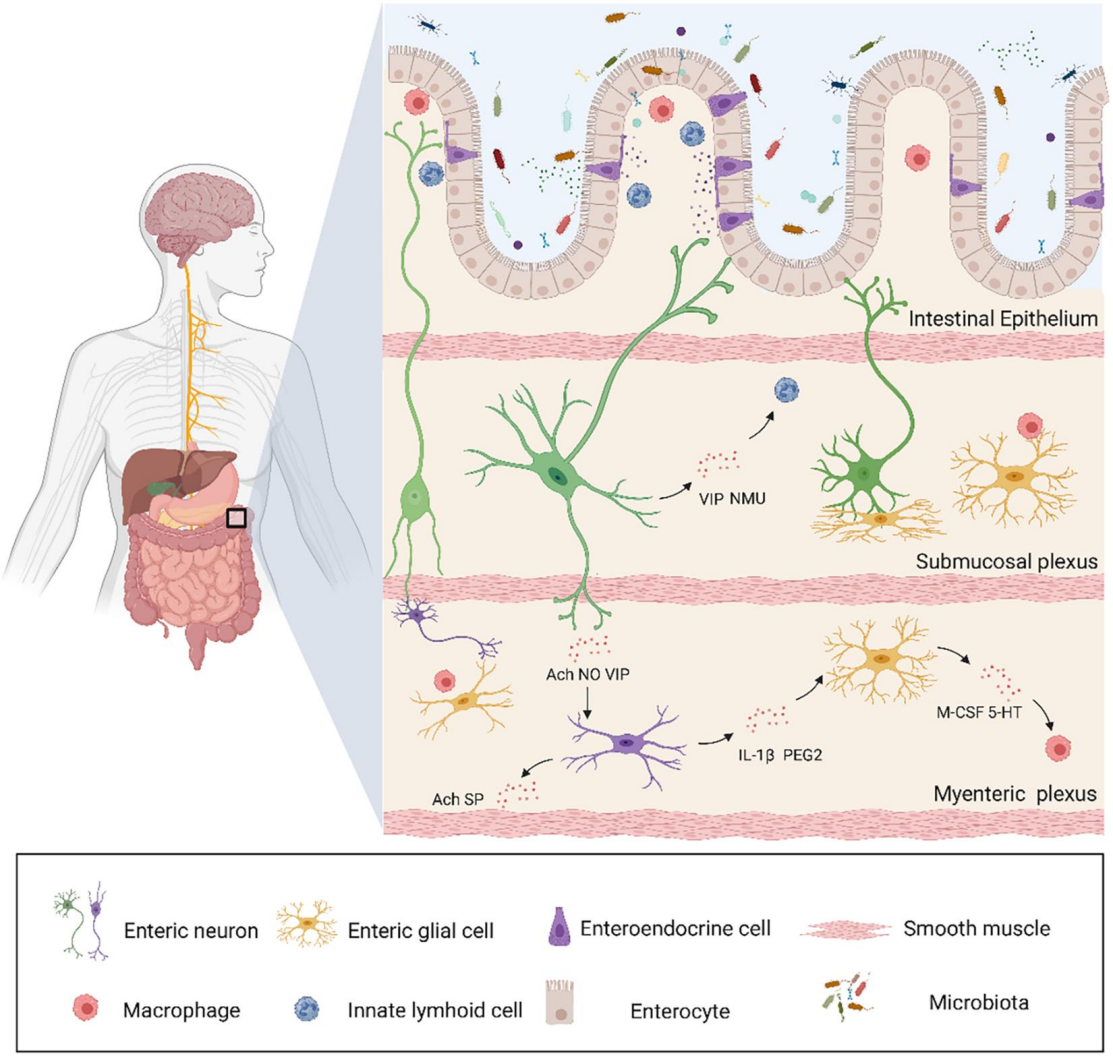
This microbiota–immune cell–enteric neuron cell interaction reveals the mechanism of enteric nervous system. Enteroendocrine cells, innate lymphoid cells, and macrophages in the intestinal epithelium are stimulated by the microbiota and release signaling factors for further action on enteric neurons. When the enteric neurons projecting to the submucosal and myenteric plexus receive the signals, they are able to activate endocrine and neural activities in these regions. Following synaptic transmission via interneurons, inhibitory or excitatory neurons are activated and regulate intestinal motility, immune responses, and local blood flow changes. During these processes, enteric glial cells tightly surround and generate bidirectional communication with enteric neurons, regulating intestinal reflex activity and maintaining normal intestinal function. VIP, vasoactive intestinal peptide; NMU, neuromodulatory peptide U; Ach, acetylcholine; IL-1β, interleukin-1β; PEG2, prostaglandin E2; M-CSF, macrophage colony-stimulating factor; 5-HT, 5-hydroxytryptamine. Created with Biorender.com.

Previous studies have extensively characterized the structure and cellular composition of the ENS, revealing the vast network of enteric neurons and glial cells that form an interconnected lattice throughout the digestive tract [5–7]. The neurons and glial cells of ENS form an extensive network that extends through the layers of the small and large intestine (Figure 1). Specifically, the myenteric plexus of the ENS is located between the longitudinal and annular layers of smooth muscle and plays a crucial role in motor control [8]. Meanwhile, the submucosal plexus is situated within the dense connective tissue of the submucosal layer, beneath the inner epithelial layer of the intestine, and is primarily involved in regulating secretion and local blood flow [9]. This ENS network is structurally and functionally distinct from CNS pathways, whose innervation reaches the intestine through the mesenteric and vascular systems. One of the remarkable features of the ENS is the variety of neurotransmitters used by its enteric neurons [10]. These neurotransmitters define the functional roles of the different enteric neurons and can be classified into several categories based on their chemical makeup. Acetylcholine is a key neurotransmitter in cholinergic neurons, which are involved in excitatory signaling and muscle contraction [11]. Neurons that secrete Substance P are associated with nociception and the modulation of pain signals within the gut [12, 13]. Enkephalin-containing neurons, which express ceruloplasmin, play an important role in controlling motility and regulating pain [14, 15]. Nitric oxide and vasoactive intestinal peptide (VIP) are found in inhibitory motor neurons, and their release helps to relax smooth muscles and promote the dilation of blood vessels [16–18]. Additionally, β-Nicotinamide adenine dinucleotide is another important inhibitor, contributing to the fine-tuning of motor function within the gut [19]. However, the neurotransmitters from neurons in the ENS differ in their roles depending on the anatomical plexus or cell of origin. The acetylcholine released by the enteric neurons of the myenteric plexus primarily binds to M3-type cholinergic receptors on the smooth muscles of the intestine, promoting smooth muscle contraction and enhancing intestinal peristalsis [8, 11]. In contrast, the acetylcholine released by the enteric neurons in the submucosal plexus affects the local neural circuits in the intestine and regulates sensitivity to stimuli, such as regulating the secretion of intestinal glands and transmitting pain responses [9, 11]. Although substance P secreted by sensory neurons typically participates in the transmission of pain signals by activating NK1 receptors when the intestine is stimulated, substance P secreted by motor neurons can promote retrograde peristalsis of the intestinal smooth muscle [13].

In conclusion, the ENS is an autonomous yet interconnected system capable of regulating the gastrointestinal tract independently of the CNS, yet continuously communicating with it. Through its complex neural network and its diverse neurotransmitter systems, ENS maintains intestinal homeostasis and influences the development of intestinal diseases.

## Phenotypes of enteric neuropathies in IBD patients

As a heterogeneous disease, the pathogenesis of inflammatory bowel disease (IBD) has not been fully clarified, but it is closely associated with genetic predisposition, environmental factors, microbiome, and the immune system [20–23]. With the advancement of research into genetic predisposition, numerous genome-wide association studies have identified multiple genetic loci associated with IBD, including NOD2, IL23R, ATG16L1, IRF1, GBP5, ARFRP1, etc. [24–29]. For example, the dysfunction of NOD2 can impair the differentiation of intestinal immune cells and inflammatory responses, while IL23R can influence the secretion of intestinal proinflammatory cytokines through IL-23/Th17 pathway [28, 29]. In IBD patients, the abnormal expression of these genes may impair the intestinal immune regulation function, leading to dysregulation of the inflammatory response and subsequently damaging enteric neurons and glial cells [23, 30, 31].

Based on studies examining the prevalence and risk factors of IBD, environmental factors also constitute significant determinants of IBD susceptibility and severity, including dietary habits, lifestyle, environmental pollution, psychological stress, etc. [32–34]. Some studies have confirmed that psychological stress has an important influence on the progress of IBD, and some stressful life events can even directly affect intestinal inflammation through ENS [35–38]. A study by Schneider *et al.* has confirmed that the glucocorticoid level continues to increase due to prolonged psychological stress, and ENS plays a key role in mediating this chronic stress to aggravate intestinal inflammation [39]. This study reveals that the continuous increase of glucocorticoid level drives the production of inflammatory subsets of intestinal glial cells, which promote monocyte- and TNF-mediated inflammation through colony stimulating factor 1 (CSF1). Concurrently, it induces immature transcription in enteric neurons and acetylcholine deficiency through transforming growth factor-β2, thereby causing impaired intestinal motility. Similar studies have also confirmed the role of the ENS as a relay station between psychological stress and intestinal inflammation during the development and progression of IBD [40–42]. With aging, a series of changes occur in the intestine, such as immune decline, imbalance of intestinal microflora, ENS degeneration, and so on, which may lead to inflammatory reaction [43–45]. The ENS is plastic, and its functional decline during aging is primarily manifested as age-related degeneration and death of enteric neurons [46]. Although similar studies mostly focus on the changes of intermuscular neurons, the number of submucosal neurons and intestinal glial cells has also been confirmed to decrease during aging [47–49]. During the aging process, enteric neuronal function, neurotransmitter level and intestinal structure undergo gradual alterations, ultimately leading to intestinal functional decline and increased susceptibility to diseases such as IBD [43, 46].

The phenotypes of enteric neuropathies in IBD patients encompass a spectrum of neural functional alterations, including heightened neural excitability, enhanced synaptic vulnerability, nerve degeneration, and diminished inhibitory neuromuscular signaling [50]. During active phases of IBD, these abnormalities are often exacerbated with structural and chemical changes in the ENS, which result in considerable neuronal damage or remodeling [51]. In turn, these changes can contribute to heightened immune reactivity, which further complicates disease progression. However, the maintenance of intestinal homeostasis is intricately dependent on a functional and coordinated interaction between the ENS, the immune system, and the intestinal microbiome [52]. In recent years, experimental evidence has increasingly indicated that disruptions in these interactions may underlie the pathogenesis of IBD and contribute to abnormalities in intestinal functions [51–53]. Linden *et al.* have demonstrated that in the context of experimental colitis, intermuscular and submucosal neurons exhibit increased excitability during the progression of colonic inflammation, which correlated with progressively worsening of intestinal function under inflammatory conditions [53]. Furthermore, according to the research results of Krauter *et al.*, inflammation in a mouse model of trinitrobenzene sulfonic acid-induced colitis leads to synaptic vulnerability via a presynaptic mechanism [54]. Interestingly, this process did not involve an incremental increase in the number of synaptic contacts per neuron, but rather enhanced neurotransmitter release and sustained activation of protein kinase A in nerve endings [54]. Such alterations suggest a complex mechanism by which inflammation disrupts normal synaptic function, contributing to the pathophysiology of the disease. Beyond synaptic dysfunction, several studies have shown that intestinal inflammation in experimental colitis results in the demise of enteric neurons, with subsequent damage to nerve fibers leading to structural abnormalities in the intestinal barrier [55–58]. These structural changes exacerbate the barrier dysfunction that is characteristic of IBD, further perpetuating the cycle of inflammation and damage. Notably, the attenuation of inhibitory neuromuscular signaling, and in some cases the complete loss of inhibitory innervation, has also been observed in experimental colitis models [59–61]. These findings highlight the critical role of the ENS in maintaining the delicate balance of intestinal function and motility, which is disrupted during active disease. Similar alterations in enteric neurophysiology have been observed in human IBD patients, further solidifying the connection between enteric neuropathy and the functional impairments seen in IBD [62]. The ENS of patients with IBD displays various lesion phenotypes and disrupts the normal physiologic function of the intestine with motility dysfunction. The plasticity and neuropathy of ENS lead to the intestinal neurophysiological changes, and play a critical role in the pathogenesis of IBD [62]. In summary, the interaction between the ENS, immune system, and intestinal microbiota is crucial for maintaining intestinal health. Disruption of this complex interaction leads to a cascade of neural and immunological abnormalities that drive the pathophysiology of IBD, affecting not only neuronal function but also the overall motility and barrier integrity of the intestine. Understanding these processes at a deeper level offers important insights into the mechanisms of IBD and potential therapeutic targets to restore normal ENS function and intestinal homeostasis.

## Interaction of neuronal cells of different secretory types with peripheral immune cells in the ENS

The development of the ENS is a complex physiological process regulated by various factors, such as genetics, environment, and hormone levels. At different ages, the development and function of the ENS exhibit distinct characteristics [63]. The development of the ENS typically begins in the fourth week of embryonic development, and the most enteric neurons and glial cells originate from the neural crest cells at the vagus nerve level within the neural tube [63, 64]. These neural crest-derived progenitor cells embark on a journey of migration and differentiation, ultimately settling in the developing intestine to constitute the ENS [64]. These enteric neurons form a network through synaptic connections, enabling the intestine to develop autonomous neural activity. After birth, the development of the ENS enters a more active phase as synaptic connections between enteric neurons increase, the gut microbiota exerts its influence, and the immune system matures [63]. The ENS begins to independently regulate basic intestinal movements (such as peristalsis and secretion) and its interactions with intestinal immune cells gradually strengthen, promoting the normal development of intestinal function [64]. By adulthood, the structure and function of the ENS have matured and become relatively stable. At this stage, the ENS can independently and efficiently regulate various physiological functions of the intestine, such as the digestive process and stress responses. Additionally, the brain–gut axis regulation between the ENS and the central nervous system becomes more refined, enabling the ENS to precisely receive and feedback information to the brain [63]. However, with further aging, the ENS undergoes certain degenerative changes, specifically manifested as the degeneration of intestinal neurons, impaired intestinal barrier function, and reduced levels of neurotransmitters [63, 64].

Enteric neurons in the ENS are classified into different types based on their function and the neurotransmitters they release, including sensory neurons, motor neurons, and interneurons [65]. Ensheathed by enteric glial cells, enteric neurons extend their axons and dendrites into various layers of the gut wall, including the external muscle layers, the submucosa, and the mucosa [65, 66]. The cell bodies of these neurons are primarily located in two major ganglia: the myenteric ganglia, which are situated between the circular and longitudinal muscle layers, and the submucosal ganglia, which lie within the submucosal layer of the intestine [66]. Furthermore, enteric neurons are interconnected with other neural circuits through the vagus nerve, allowing bidirectional communication that integrates both local and central signals [63]. The interaction between neuronal cells and peripheral immune cells in the ENS is complex and multifaceted, primarily occurring through the secretion of neurotransmitters, cytokines, and various signaling pathways [52]. These factors influence the activation and recruitment of immune cells in the intestinal microenvironment. For example, different subsets of immune cells in the intestine, such as macrophages and dendritic cells, respond to neuronal signaling by expressing specific neurotransmitter and neuropeptide receptors [67]. Conversely, intestinal neurons express receptors for cytokines and other immune signals, allowing them to respond to local inflammatory stimuli [68]. The crosstalk between neurons and immune cells is essential for maintaining intestinal homeostasis and plays a central role in regulating inflammatory responses within the intestine [67, 68]. Several studies have established the presence of functional neuron-immune cell interactions, which play a critical role in regulating intestinal inflammation [67–70]. These interactions are critical for both the initiation and resolution of inflammation; disturbances in this balance can contribute to the pathogenesis of IBD [71]. Through the complex interplay of neuronal, immune, and epithelial cells, the ENS helps to modulate immune responses, ensuring that the intestine can both defend against pathogens.

### Innate lymphoid cells

Innate lymphoid cells (ILCs), also known as intrinsic lymphoid cells, are a group of innate immune lymphocytes (such as ILC1, ILC2, and ILC3). These cells can respond rapidly to infections and stress signals without the need for prior sensitization, enabling them to play an essential role in the early detection and response to pathogens. The specific functions of ILCs are determined by their diverse array of cytokines and transcription factors, which vary depending on the ILC subtype and tissue context [71]. For instance, ILC1s predominantly produce interferon-gamma (IFN-γ), ILC2s secrete interleukin-5 (IL-5) and IL-13, while ILC3s are known for their production of IL-17 and IL-22. Intestinal ILCs, which are fast-responding and highly abundant, serve a “sentinel” function in preserving the integrity of the intestinal barrier [72]. ILCs are involved in regulating the balance between pro-inflammatory and anti-inflammatory signals, which is essential for intestinal homeostasis and protection from inflammatory diseases like IBD.

VIP, a hormone meticulously secreted by enteric neurons, is involved in a large number of ENS-mediated intestinal functions, and it is closely associated with ILCs during the intestinal immune response [68, 73]. Specifically, Seillet *et al.* have demonstrated that intestinal ILC3s, a subset of ILCs, can activate a population of enteric neurons that express VIP [74]. The high expression of VIPR2 on ILC3s promotes tissue-specific production of IL-22, a cytokine that modulates intestinal barrier function and promotes anticipatory mucosal immunity [73, 74]. Conversely, as shown in experimental models of colitis, the disruption of VIP signaling interferes with the production of IL-22 in ILC3, consequently increasing the inflammatory susceptibility of the intestine [75, 76]. Beyond the VIP-ILC3 interaction, additional research has expanded our understanding of the complex communication network between the ENS and ILCs. Notably, the work of Ibiza *et al.* has shown that enteric glial cells are adjacent to ILC3 and project into ILC3 aggregates, which serve as part of the glial–ILC3–epithelial cell unit orchestrated by neurotrophic factors [77]. In this setting, enteric glial cells express neurotrophic factors downstream of the p38 MAPK/ERK-AKT cascade in a Myd88-dependent manner [77]. This signaling promotes the activation of innate immune responses, particularly the production of IL-22, which is crucial for intestinal defense [77, 78]. These results suggest that interactions between enteric neurons, enteric glial cells, and IL-3 are involved in the modulation of the intestinal immune environment [73, 77, 78].

In addition to VIP and glial-derived signals, cholinergic neurons in the intestine have also been identified as key regulators of ILC function [79]. According to several studies, intestinal cholinergic neurons exert local neural regulation of ILCs by releasing the neuromodulatory peptide U (neuromedin U, NMU) [79–81]. The NMU is a key cholinergic neuropeptide that enhances ILC2 responses, with its receptor, NMUR1, being selectively enriched in ILC2 [80]. Recent studies have demonstrated that NMU expression levels are elevated in experimental colitis, and the NMU-NMUR1 signaling axis activates ILC2 in a MyD88-dependent manner [79, 82]. Notably, NMU-NMUR1 signaling drives the production of type 2 effector molecules and the release of the tissue-protective factor amphiregulin [83, 84]. Taken together, these findings illustrate a sophisticated network of interactions between ILCs and ENS, which together contribute to the modulation of the intestinal immune environment. Disruptions to these neuroimmune interactions can lead to the dysregulation of intestinal immune responses, contributing to inflammatory diseases like IBD. Understanding the precise molecular mechanisms behind these interactions may open up new therapeutic avenues for treating gastrointestinal disorders.

### Macrophages

Intestinal macrophages are a vital component of the immune system, and play key roles in immune surveillance and the maintenance of intestinal homeostasis as a diverse population of innate immune cells [85]. These macrophages are essential for both defending the body against pathogens and maintaining a balanced and functional immune response within the intestinal environment. Gabanyi *et al.* have found that macrophages in the gut exhibit a high degree of gene-expression specialization, and have different functional and morphological characteristics in different localizations [86]. This specialization is integral to the macrophages’ ability to adapt to the specific needs of the intestinal microenvironment. Lamina propria macrophages are predominantly found in the connective tissue beneath the epithelial layer of the intestine. These cells are marked by the expression of the receptor CX3CR1 and preferentially express a pro-inflammatory phenotype, characterized by elevated levels of IL1b and IL2b [86–88]. In contrast, muscularis macrophages (MMs) are more proximal to the myenteric plexus and exhibit a tissue-protective phenotype, characteristically expressing wound healing genes such as Mrc1 and Chi3l3 [86, 89].

In recent years, several studies have shown that macrophages play a key role in the cholinergic anti-inflammatory pathway in the intestine that significantly impacts intestinal health and disease states [90–93]. This process benefits from the close contact between enteric neurons and immune cells (especially MMs), which may produce a complex set of interactions between neurotransmitters, cytokines, and hormones [94–96]. For example, enteric neurons can influence the activity of intestinal macrophages through neurotransmitter secretion, such as acetylcholine, which can enhance the anti-inflammatory effect of intestinal macrophages and promotes their adaptive immune response [95]. This interaction underscores the importance of neural-immune crosstalk in modulating immune responses in the intestine. According to Viola *et al.*, intestinal macrophages play an important role in the development and maturation of the ENS, constituting a specific macrophage population that maintains intercellular communication with the ENS and meets the demands of the ENS ecological niche through adaptive changes in its phenotype and transcriptome [97]. MMs are involved in postnatal neuronal refinement, including the phagocytosis of neuronal synapses and cell bodies, as well as the dynamic regulation of neuronal density [97, 98].

Furthermore, the vagal anti-inflammatory response, often referred to as the “inflammatory reflex,” illustrates the complex interplay between the nervous system and immune system in the intestine [99]. The vagus nerve, when activated during inflammation, helps mediate a systemic anti-inflammatory response, which has been demonstrated in models of sepsis and postoperative intestinal obstruction [100, 101]. However, during conditions such as intestinal inflammation, the dynamics of this system can be disrupted. In this context, monocytes and immature macrophages are recruited in large numbers and retained in the mucosal compartment, where they secrete high levels of inflammatory mediators [102, 103]. These inflammatory mediators, including tumor necrosis factor alpha (TNF-α), IL-1, and IL-6, contribute to tissue damage and affect intestinal motility and secretory function by disrupting neuronal signaling in response to inflammation [104, 105]. Interestingly, it has been shown that macrophages enriched in the vicinity of the intestinal epithelium are sufficient to respond rapidly to pathogens, migrating into the intestinal lumen to restrict the number of bacteria breaching the epithelial barrier [106]. This rapid response is vital for preventing infections and maintaining intestinal integrity. However, in comparison to healthy individuals, the dysfunction of ENS in IBD patients is closely related to the abnormal activation of macrophages, which contributes to chronic inflammation and immune imbalance in the intestine [107, 108]. In conclusion, intestinal macrophages are central to both the immune surveillance of the gut and the maintenance of intestinal homeostasis. Dysregulation of these macrophages, particularly in the context of diseases like IBD, can lead to chronic inflammation and impaired intestinal function.

### Muscle cells

As an important component of the autonomic nervous system, the ENS regulates a variety of intestinal functions, including motility, secretion, and blood flow [1, 5–7]. Intestinal smooth muscle cells are the executors of intestinal motility and are responsible for intestinal contraction and relaxation [109]. The efficiency of these processes is regulated by signals from the ENS, highlighting the critical interaction between neural control and smooth muscle activity [110]. The interactions between the ENS and intestinal smooth muscle cells have an important impact on the normal function of the intestinal tract and the development of related diseases [109–111]. An animal experiment by Graham *et al.* showed that early muscle differentiation occurs after the arrival of migrating enteric neural crest-derived cells, suggesting a spatial and temporal correlation between the ENS and the development of intestinal smooth muscle [109]. This finding indicates that the ENS is not merely an accessory to muscle function but plays a crucial role in shaping the development of the gastrointestinal system. During embryonic development, mechanical stresses generated by intestinal smooth muscle cells play a role in shaping the developing ENS [112]. This highlights a dynamic, reciprocal relationship between the mechanical and neural components of the intestine, where the activity of one system can influence the growth and function of the other.

In addition, chemical signaling pathways also play a significant role in coordinating ENS-smooth muscle communication. One such pathway involves gamma-aminobutyric acid (GABA), a neurotransmitter traditionally associated with inhibitory signaling in the central nervous system. A recent study by Liu *et al.* has demonstrated that the GABAergic signaling pathway between the ENS and intestinal smooth muscle has an impact on intestinal immune defenses [113]. Their transcriptomic analysis has shed further light on the molecular underpinnings of this communication. The neuropeptide FLP-6, expressed and secreted by intestinal smooth muscle cells, acts downstream of intestinal GABAergic signaling and functions as a paracrine signaling molecule within the intestinal epithelium [113]. Similar studies have confirmed the important role of the enteric neuron-smooth muscle cell-intestinal intrinsic immune signaling axis in intestinal immune regulation, and revealed the influence of the nervous system in intestinal defense, immunity, and inflammatory responses [114, 115]. Despite these insights, the relationship between the ENS and intestinal immunity remains complex. Different neurotransmitters and their respective receptors may have opposing roles in immune responses [113]. For example, while GABAergic signaling generally supports immune modulation, other neurotransmitters and their receptors can exert contrasting effects, potentially leading to imbalanced or incomplete immune responses. Even different receptors for the same neurotransmitter may have opposite effects on the enteric neuron-smooth muscle cell-intrinsic intestinal immune signaling axis [116]. Such complexities can lead to incomplete immune responses and affect the determination of the role of the interaction of GABAergic neurons and smooth muscle cells on intestinal immunity [113, 116]. These complexities make it challenging to fully decipher the precise mechanisms by which the ENS influences intestinal immune responses, and underscore the need for further research to clarify the roles of different neurotransmitter systems in gut defense.

## Effects of glial cells in the ENS on immune cells

Deriving from neural crest cells, enteric glial cells (EGCs) eventually migrate and integrate into the myenteric plexus and submucosal plexus of the ENS, where they support and interact with enteric neurons [117, 118]. Astrocytes and microglia are the predominant neuroglial cell types in the gut, and they play important roles in neuronal protection and immune regulation [104]. Structurally, EGCs are characterized by their long, branched processes, often surrounding enteric neurons and extending throughout the intestinal wall [117, 119]. Functionally, EGCs are components of the ENS, providing support to neurons, regulating neurotransmitter signaling, and modulating immune responses [120]. They form a complex network in conjunction with enteric neurons, which play important roles in neuronal protection and immune regulation. EGCs can regulate the expression of neuropeptides, neurotransmitter receptors, and cytokines, which help maintain the intestinal epithelial barrier and modulate immune cells such as mast cells and macrophages [117, 119]. According to several studies, some pro-inflammatory factors associated with IBD, including interleukin (IL)-1β, TNF-α, and IFN-γ, can activate EGCs and transform them into reactive glial cells, which disrupt the supportive role of EGCs [121–123]. In addition to the neuroglial response to inflammation, patients with CD have been observed to exhibit reduced numbers or functional defects of enteric glial cells in the intestinal submucosal plexus [124]. Existing studies have shown that defects in the enteric glial network are often observed in patients with Crohn’s disease, including damage of nerve fiber, hypertrophy of neuronal cell bodies, and enteric glial cell hyperplasia [124–126]. These pathologic manifestations can affect structure and function within the ENS, resulting in alterations in bidirectional communication through the gut–brain axis [127, 128]. These changes in EGCs can cause an increase in the expression of immune cell-related biomarkers, which can ultimately lead to the onset and recurrence of IBD [129].

Additionally, the findings of Dora *et al.* have shown the presence of a specific type of macrophage in the enteric ganglion, which is able to act as a pro-inflammatory factor into EGCs in experimental colitis [130]. Another study has demonstrated that upon inflammation-induced stimulation, specifically by IL-1β, EGCs of the myenteric plexus can activate myenteric macrophages by producing macrophage colony-stimulating factor (M-CSF) in a connexin 43-dependent manner [131]. This suggests the potential role of EGC-macrophage interactions as pro-inflammatory instigators in the progression of intestinal inflammation. Bidirectional communication between EGCs and immune cells also plays a key role in maintaining intestinal immune homeostasis, including the coordination of intestinal motility and regulation of mucosal barrier immunity [129]. EGCs engage in intricate signaling cascades with immune cells resident in the gut, particularly dendritic cells, macrophages, and T cells, by releasing serotonin and its metabolites [132]. Serotonin, as an important neurotransmitter, regulates immune cell migration, activation, and cytokine secretion, thereby influencing local immune responses [133]. In turn, activation of immune cells and subsequent inflammatory responses can influence the function of EGCs. Immune cells in the intestinal immune system regulate the level of serotonin secretion from EGCs by secreting cytokines (e.g. IL-4, TNF-α, etc.) [134]. Taken together, EGCs serve as connective and regulatory intermediaries between immune cells, the intestinal epithelial barrier, and enteric neurons. The effects of activation of EGCs on enteric neurons and immune cells remain to be further investigated, which will be beneficial for understanding the development of IBD and identifying its potential therapeutic targets [124, 135].

## Interactions between EGCs and enteric neurons

Enteric neurons, the sentinels of the intestinal milieu, execute a myriad of functions within the intestinal tract. They encompass sensory and motor control, as well as regulation of the intestinal environment, while EGCs collaborate with these neurons to maintain normal intestinal function [136, 137]. The bidirectional communication between enteric neurons and glia regulates intestinal reflexes. EGCs monitor multiple forms of neural activity through neurotransmitter receptor signaling. Recent studies have shown that interactions between enteric neurons and EGCs influence the output of enteric neural circuits, which facilitates the exploration of the mechanisms of normal enteric circuitry action as well as the development of dysfunction in intestinal disorders [138–140]. Stavely *et al.* have demonstrated the reinnervation potential of post-mitotic enteric neurons through selective neuronal tracing in BAF53b-Cre mice [117]. In their animal experiments, isolated enteric neurons regenerated neural synapses *in vitro*, with the complexity and orientation of these synapses being influenced by interactions with EGCs.

Meanwhile, EGCs exert a supportive role in enteric neurotransmission via astrocyte-like mechanisms, including supplying neurotransmitter precursors, regulating neurotransmitter availability, orchestrating oxidative stress, and facilitating neurogenesis [65, 141, 142]. EGCs help maintain homeostasis in the ENS by providing neurotransmitter precursors, which are essential for the synthesis of neurotransmitters such as acetylcholine [65]. EGCs regulate the availability and reuptake of neurotransmitters, ensuring optimal signaling between enteric neurons [141, 142]. In addition, EGCs are involved in the coordinated response to oxidative stress, which would otherwise lead to neuronal damage and dysfunction [142, 143]. To demonstrate the direct protective effect of EGCs on enteric neurons, Abdo *et al.* have reported that EGCs prevented oxidative stress-induced damage to enteric neurons by releasing reduced glutathione, as shown in co-culture experiments with EGC lines and primary cultures of the ENS [143]. EGCs not only support neuronal repair and regeneration and innervation, but also contribute to the plasticity of ENS [117, 119]. It is noteworthy that EGCs exhibit remarkable plasticity, particularly in response to injury or pathological conditions. Interestingly, EGCs can differentiate into enteric neurons following chemically induced tissue injury, inflammation, or infection [144, 145]. This regenerative capacity underscores the potential of EGCs for the repair and functional restoration of the ENS following damage. In addition, active signaling mechanisms between EGCs and enteric neurons play an integral role in regulating gastrointestinal reflexes [128]. These signaling processes help maintain normal intestinal function, and, in some cases, can trigger neuroinflammatory processes that lead to long-term dysfunction [146].

Neuropeptides secreted by intestinal neurons (e.g. substance P, VIP, etc.) may also affect the function of EGCs during intestinal inflammation [147]. For example, substance P promotes glial cells to secrete more pro-inflammatory factors by acting on the receptors on the surface of EGCs, forming a positive feedback loop that further exacerbates the inflammatory response [148]. It has been demonstrated that EGCs may cause abnormalities in enteric neuron function by altering signaling interactions with enteric neurons [149]. The development of reactive gliosis is one of the main features of intestinal neuroplasticity, usually triggered by noxious stimuli, such as inflammation, as well as intense activity of enteric neurons [139, 150]. In this context, the development of reactive gliosis can initiate a self-perpetuating process and ultimately lead to neuroinflammation and subsequent abnormalities in neuronal plasticity and function [128]. During neurogenic inflammation, active glial signaling has a strong purinergic component that promotes reactive gliosis, neuronal death, and neuromuscular functional changes [57, 139, 150]. When this active signaling mechanism is activated, ATP released by glial cells acts on neuronal P2X7 receptors to alter neuronal survival and function [57, 150]. Interestingly, a significant nitric oxide component is present in pathological neuron-glia signaling during inflammation, whereas normal neuron-glia signaling is absent [149]. As a pro-inflammatory mediator, nitric oxide, produced by glial cells during acute inflammation, can interact with normal signaling mechanisms to promote ATP release and subsequent neuroinflammation [150]. In conclusion, enteric glia can alter neuronal plasticity and function through active signaling mechanisms and processes that regulate the secretion of neuroactive compounds.

## ENS and colorectal cancer

It has been shown that the ENS plays a significant role in modulating the inflammatory processes within the intestine, primarily through the release of neurotransmitters and the regulation of immune responses [39, 52]. Enteric neurons can regulate the levels of inflammation by secreting cytokines and neuropeptides and interacting with immune cells in the intestine. These neurons interact with immune cells, including macrophages, dendritic cells, and T-cells, to either promote or resolve inflammation depending on the context. Research has increasingly focused on how these interactions might contribute to the pathogenesis of gastrointestinal diseases, including IBD and colorectal cancer [151–153]. It has been indicated that specific immune cells and cytokines can facilitate the progression from intestinal inflammation to cancer [42]. For instance, IL-22, a cytokine produced by immune cells such as Th17 cells, has been shown to act specifically on epithelial cells [154]. IL-22 can induce the phosphorylation of signal transducer and activator of transcription 3, a key signaling molecule involved in cell proliferation and survival [155, 156]. Chronic activation of this pathway in the epithelial cells of the colon can lead to uncontrolled cell division, creating a microenvironment conducive to the development of colon cancer in the context of persistent inflammation in patients with IBD [123]. Furthermore, other research has outlined the mechanisms through which chronic intestinal inflammation fosters a tumorigenic environment [152, 153]. Chronic inflammation results in ongoing tissue damage, which can lead to cellular dysfunction, genetic mutations, and significant alterations in the tumor microenvironment [152]. This damage acts as a driver of colorectal cancer development through processes like apoptosis resistance, accumulation of genetic mutations, and changes in the extracellular matrix [153].

The role of the ENS in the development of colorectal cancer has been increasingly acknowledged, with several studies pointing to its involvement in perineural invasion, neurogenesis, and paracrine secretion between enteric neurons and cancer cells [157, 158]. Interestingly, the number of neurons in the intestine tends to increase in patients with colorectal cancer, which may be related to changes in the tumor microenvironment [158]. This increase in neuronal density is thought to be driven by the changes in local signaling factors, which may create a pro-tumorigenic environment [159]. Some neurotransmitters secreted by enteric neurons, including norepinephrine, acetylcholine, and netrin-1, have the capacity to stimulate tumor proliferation and metastasis by binding to receptors on tumor cells [157, 159]. These neurotransmitters can activate signaling pathways that accelerate cell division, survival, and migration, thereby contributing to tumor progression [157]. In addition to enteric neurons, EGCs, once considered merely supportive cells in the ENS, have been shown to be integral to the colon cancer microenvironment in recent years [160–162]. Recent studies have highlighted the integral role of EGCs in promoting tumor growth, particularly in the context of colorectal cancer [163, 164]. Yuan *et al.* designed experiments on glial fibrillary acidic protein-viral thymidine kinase transgenic mice and revealed that GFAP + enteric neuroglial cells play a key pro-tumorigenic role in early colorectal cancer development [163]. These glial cells interact with tumor cells and facilitate the activation of colon cancer stem cells, which are thought to be responsible for the initiation, maintenance, and metastasis of cancer [162, 163]. EGCs promote the activation of colon cancer stem cells through the epidermal growth factor receptor (EGFR) and extracellular regulated protein kinases signaling pathways, which enhance the expansion and tumor-generating capacity of colon cancer stem cells [163, 165]. Upon activation and acquisition of a pro-tumorigenic phenotype, EGCs are able to stimulate colon cancer stem cell-driven tumorigenesis through a PGE2/EP4/EGFR-dependent pathway [162].

**Figure 2:**
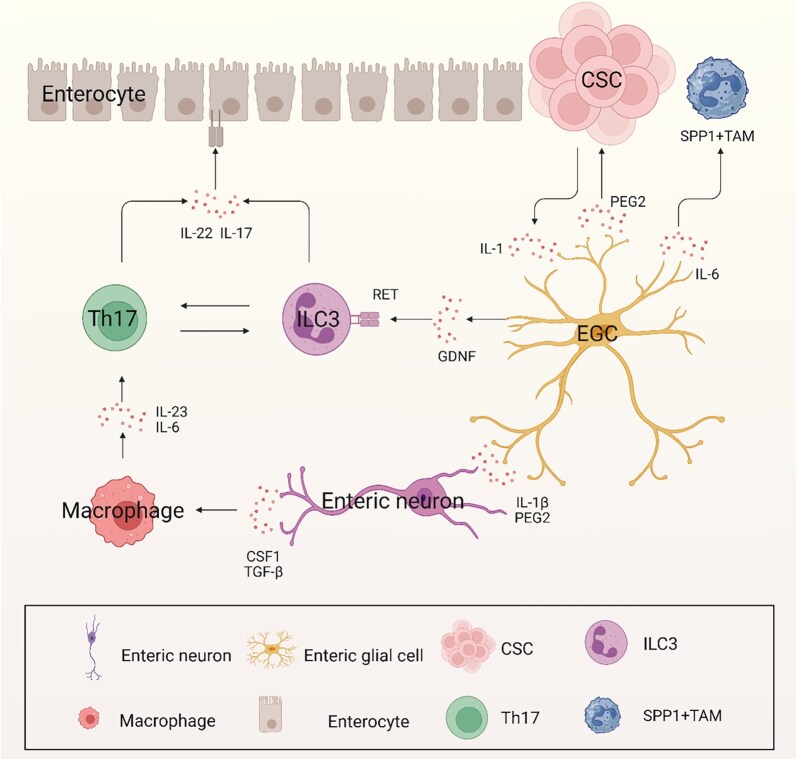
The enteric neurons–CSC–immune cells–EGCs interactions reveal the pathogenesis of enteric nervous system in inflammatory bowel disease and colorectal cancer. EGCs, enteric glial cells; CSCs, colon cancer stem cells; ILC3, group 3 innate lymphoid cell; Th17, T helper cell 17; SPP1+ TAM, secreted phosphoprotein 1 tumor-associated macrophages; PEG2, prostaglandin E2; IL, interleukin; GDNF, glial-derived neurotrophic factor; ILC3, innate lymphoid cells; CSF1, colony-stimulating factor 1; TGF-β, transforming growth factor-β. Created with Biorender.com.

Furthermore, numerous recent studies on intestinal inflammation have revealed IL-1-mediated EGC reactivity and its impact on immune cell regulation [122, 166]. When activated by neuroglial IL-1R signaling, EGCs exhibit a reactive cellular phenotype and drive their functional transformation into cells that support tumorigenesis [122]. Thereafter, IL-1-triggered EGC-IL-6 release plays a critical role in the regulation of tumor growth and SPP1+ TAM differentiation [166, 167]. However, a recent study has suggested that a positive feedback loop emerges between EGCs and tumor-associated macrophages in colorectal cancer [167]. This feedback loop, observed in both *in vivo* and *in vitro* models, suggests that neuroglial cells and immune cells interact in a dynamic manner to enhance the malignancy of the tumor [167]. In this context, EGCs are not just passive components of the intestinal microenvironment, but rather active modulators of cancer progression, interacting with both the immune system and tumor cells to drive the development and growth of colorectal cancer. In conclusion, the ENS, including its neurons and glial cells, plays a multifaceted role in both the inflammatory response and cancer progression in the intestine. From regulating immune cell activity to directly influencing tumor growth through neuroimmune interactions, the ENS is an important factor in the development of colorectal cancer, particularly in the setting of chronic intestinal inflammation [39, 122, 152, 153]. The interactions between enteric neurons, colon cancer stem cells, immune cells, and EGCs are illustrated in Figure 2. Understanding these complex interactions provides valuable insights into potential therapeutic strategies for treating both IBD and colorectal cancer.

## Novel treatments for IBD targeting ENS and future perspectives

The ENS plays an important role in the pathogenesis of IBD, a condition characterized by chronic inflammation of the gastrointestinal tract. The complex interactions between the enteric neurons, EGCs, and immune cells contribute to the onset and progression of IBD [62, 124, 143]. As a result, the ENS has become a key focus of research for novel therapeutic strategies aimed at managing IBD. In recent years, innovative ENS-based therapeutic approaches, particularly those involving electrical neuromodulation (ENM), have proven effective in treating a variety of gastrointestinal disorders, including dyspepsia, constipation, IBD, and so on [168]. These approaches offer new perspectives on managing the condition by modulating the neural and immune responses that underlie intestinal inflammation. ENM refers to the use of electrical impulses to modify the activity of neural circuits in the gastrointestinal system, which can have both direct and indirect effects on intestinal motility, inflammation, and pain perception [168, 169]. In preclinical research, the potential benefits of ENM have been proved by experimental models. Moreover, neuromodulators, which are substances that alter nerve activity, have shown promise in modulating neurotransmitter release within the ENS and thereby improving intestinal function. By affecting the release of key neurotransmitters such as serotonin, acetylcholine, and nitric oxide, neuromodulators can help restore normal ENS function to regulate intestinal motility, secretion, and immune response [170]. Recent research has explored the effects of various compounds, such as sodium butyrate and farrerol, on nerve conduction in the 2,4,6-trinitrobenzene sulfonic acid-induced models of colitis [170, 171]. These experimental models of colitis not only provide valuable insights into the potential of ENM therapy but also establish a new theoretical basis for neuromodulatory treatment of IBD. However, these interventions are still in the experimental stage and more clinical trials and basic research are needed to validate their safety and efficacy.

In clinical application, the ENM offers a novel approach to treating IBD, particularly by targeting the neural pathways associated with inflammation and pain. Several studies have demonstrated the potential benefits of ENM in controlling IBD-related symptoms [168, 169, 172]. By targeting the neural networks of the ENS, ENM can modulate gastrointestinal functions, potentially providing relief from symptoms of IBD and other related conditions. A key feature that underpins the use of ENM in treating IBD is the bidirectional communication between the ENS and the CNS. This communication network forms the basis for bioelectrical neuromodulation, where electrical signals are used to influence the activity of both the ENS and the CNS [169]. In addition, electrical brain nerve stimulation has been shown to suppress inflammatory responses, which is particularly important in conditions like IBD, where inflammation is a hallmark of the disease [144]. On the other hand, spinal cord stimulation has been reported to reduce diarrhea and pain, common symptoms in IBD patients [173]. These findings suggest that targeting specific neural circuits through ENM can alleviate both the inflammatory and sensory aspects of IBD. In order to assess the safety, efficacy, and long-term effects of these treatment modalities in patients with IBD, it remains necessary to understand the optimal parameters of electrical stimulation, the specific neural pathways, and the potential side effects associated with these treatments.

The Piezo1 in intestinal neurons, a recently identified mechanically activated nonselective cation channel protein, senses intestinal pressure and regulates intestinal motility [174]. However, in the context of IBD the dysfunction of Piezo1 weakens the intestine’s motor response to pressure and exacerbates inflammation [175]. Targeted activation of Piezo1+ neurons, in this case, can enhance intestinal motility and help alleviate inflammation [176, 177]. The gut–brain axis is a two-way biochemical communication pathway between the CNS and ENS, enabling a mutual influence between brain and peripheral intestinal functions [178]. Based on the regulation of gut–brain axis, vagus nerve stimulation can enhance the activity of ENS, inhibit the production of inflammatory cytokines by immune cells, and thus regulate intestinal motility and immune function [179, 180]. Although there is currently insufficient clinical evidence, combined treatments of both biologics and ENS targeted therapies provide a new idea and method for the treatment of IBD, which will be beneficial to some refractory IBD patients. In conclusion, as research progresses, these innovative therapies may offer new hope for patients suffering from IBD, especially those who do not respond well to traditional pharmacological treatments.

Although significant progress has been made in the study of ENS in recent years, several limitations persist that hinder comprehensive investigations in this area. One of the primary challenges lies in the relative scarcity of neuronal cells compared with glial cells within the ENS, which limits single-cell sequencing in ENS studies. Specifically, in human intestinal tissue, after enrichment processes, the percentage of neuronal cells rarely exceeds 4 per 1,000, making it difficult to isolate and study individual neurons. Additionally, existing methodologies for studying the ENS primarily include electrophysiological recordings, imaging techniques, and molecular biology methods. These techniques contribute valuable insights, but have limitations in spatial and temporal resolution, which makes it challenging to capture the dynamic interactions between neurons and glial cells in real-time. To address these challenges, there is growing interest in integrating high-resolution spatial transcriptomics with single-cell sequencing techniques. Spatial transcriptomics allows for the precise mapping of gene expression within intact tissues, providing a spatial context to molecular data that can uncover previously unrecognized cellular heterogeneity within the ENS. When combined with single-cell sequencing, this approach has the potential to provide a detailed, multi-dimensional view of the ENS, particularly in its involvement in disease processes like tumor development, inflammation, and neurodegeneration. In the future, relevant studies will need to incorporate knowledge from multiple disciplines, including molecular biology, spatial transcriptomics and neuroscience, to further reveal the role of ENS in pathological conditions such as inflammation and cancer.
